# Body mass index outperforms the Rotterdam criteria in defining phenotypic heterogeneity in polycystic ovary syndrome: a multicenter study from the Italian PCOS registry (PCOSOUTCOME.net)

**DOI:** 10.1007/s40618-026-02946-9

**Published:** 2026-08-28

**Authors:** Alessandra Gambineri, Silvia Federici, Elisa Maseroli, Laura Guccione, Marco Bonomi, Anna Maria Fulghesu, Roberta Giordano, Roberto Mioni, Rosario Pivonello, Laura Rotolo, Chiara Sabbadin, Carla Scaroni, Massimo Tonacchera, Herbert Valensise, Nunzia Verde, Linda Vignozzi, Paolo Moghetti, Costanzo Moretti

**Affiliations:** 1 Division of Endocrinology and Diabetes Prevention and Care, IRCCS Azienda Ospedaliero-Universitaria di Bologna, Bologna, Italy; 2 Department of Medical and Surgical Sciences (DIMEC), Alma Mater Studiorum University of Bologna, Bologna, Italy; 3 Department of Endocrine and Metabolic Diseases-IRCCS Instituto Auxologico Italiano, Milan, Italy; 4 Department of Medical Biotechnology and Translational Medicine, University of Milan, Milan, Italy; 5 Andrology and Gender Endocrinology Unit, Careggi University Hospital, Florence, Italy; 6 Department of Systems’ Medicine, University of Rome Tor Vergata, Rome, Italy; 7 Department of Surgical Science, Duilio Casula Hospital, University of Cagliari, Monserrato, Cagliari, Italy; 8 Department of Biological and Clinical Sciences, University of Turin, Turin, Italy; 9 Department of Medicine, Clinica Medica 3-Azienda Ospedaliera, University of Padua, Padua, Italy; 10 Dipartimento di Medicina Clinica e Chirurgica, Sezione di Endocrinologia, Università Federico II di Napoli, Naples, Italy; 11 Department of Medicine (DIMED), University of Padova, Padova, Italy; 12 Endocrinology Unit, University Hospital of Padova, Padova, Italy; 13 Department of Endocrinology, Pisa University Hospital of Cisanello, Azienda Ospedaliera Universitaria, Pisa, Italy; 14 Cattedra di Ginecologia ed Ostetricia, Università di Tor Vergata, Policlinico Casilino, Roma, Italy; 15 Dipartimento di Medicina Clinica e Chirurgia, Sezione di Endocrinologia, Diabetologia, Andrologia e Nutrizione, Unità di Andrologia e, Medicina della Riproduzione, Sessualità e Affermazione di Genere, Università Federico II di Napoli, Naples, Italy; 16 Department of Experimental, Clinical, and Biomedical Sciences “Mario Serio”, University of Florence, Florence, Italy; 17 Unit of Endocrinology, Diabetes and Metabolism, Department of Medicine, University of Verona, Verona, Italy

**Keywords:** Polycystic ovary syndrome, Phenotypes, Obesity, Metabolic complications, Hyperandrogenism

## Abstract

**Purpose:**

Polycystic ovary syndrome (PCOS) is a common and highly heterogeneous endocrine–metabolic disorder. The Rotterdam criteria identify four diagnostic phenotypes; however, it remains unclear whether these phenotypes or body mass index (BMI) better predict metabolic risk. The PCOSOUTCOME.net registry collects data from multiple Italian centers and provides an opportunity to compare the effectiveness of Rotterdam phenotypes versus BMI in characterizing the clinical, metabolic, and psychological heterogeneity of women with PCOS.

**Methods:**

We included 1,314 Caucasian women of reproductive age with PCOS. Participants were stratified according to Rotterdam phenotypes and BMI categories (normal weight, overweight, and obesity; and BMI < 27 kg/m² vs. ≥ 27 kg/m²). Clinical, hormonal, metabolic, gynecological, and psychological parameters were assessed and compared across subgroups.

**Results:**

No significant differences in BMI or in the prevalence of metabolic complications (dyslipidemia, glucose intolerance, hypertension, and metabolic syndrome) were observed among the four Rotterdam phenotypes, although anxiety scores were highest in phenotype A. In contrast, BMI-based stratification identified clear differences in metabolic markers: women with obesity or a BMI ≥ 27 kg/m² showed a higher prevalence of all metabolic complications, as well as higher free androgen index (FAI) and hirsutism scores.

**Conclusions:**

In this multicenter cohort, Rotterdam phenotypes failed to discriminate metabolic risk, whereas BMI-based stratification proved more informative in identifying cardiometabolic complications. Moreover, obesity in PCOS identifies a distinct hyperandrogenic phenotype. These findings underscore the need to integrate BMI into the classification of PCOS to better capture patient heterogeneity, with potential implications for metabolic assessment and risk stratification.

## Introduction

Polycystic ovary syndrome (PCOS) is an increasingly prevalent endocrine–metabolic disorder affecting approximately 10–15% of women worldwide [1]. Although hyperandrogenism and irregular menstrual cycles represent central features of the diagnostic criteria [2], the syndrome encompasses a broad spectrum of reproductive and cardiometabolic abnormalities that vary in severity and clinical presentation [3]. Growing evidence suggests that PCOS cannot be considered a single clinical entity, but rather a collection of phenotypic subtypes with distinct reproductive and metabolic risk profiles, which complicates diagnosis and necessitates individualized approaches to management [4–6]. Standard diagnostic frameworks identify different phenotypes defined by the presence or absence of hyperandrogenism, ovulatory dysfunction, and polycystic ovarian morphology (*PCOm*). The Rotterdam criteria, in particular, define four clinical phenotypes (A–D). Although many studies suggest that the hyperandrogenic phenotypes (A–C) are associated with a more severe metabolic profile compared with phenotype D [7], several investigations have reported minimal or no significant differences in metabolic parameters among the Rotterdam phenotypes, particularly when patients are stratified by weight (leanness vs. obesity) [8]. These findings suggest that BMI and adiposity may play a major role in shaping the phenotypic expression of PCOS and could potentially provide a more informative and comprehensive stratification of patients. Therefore, leveraging data from the first Italian PCOS registry, PCOSOUTCOME.net, we conducted an observational study to compare the ability of the Rotterdam phenotypic classification and BMI to characterize phenotypic heterogeneity in women with PCOS.

## Methods

### The registry PCOSOUTCOME.net

The PCOSOUTCOME.net registry is designed as a multicenter, national, observational study involving patients with PCOS. It is promoted by the Club of the Italian Society of Endocrinology (SIE) – Women’s Endocrinology. Expert centres with recognized expertise in PCOS and affiliated with the SIE Women’s Endocrinology Club were invited to participate in the registry and to enrol patients with PCOS of all ages, provided that the diagnosis was established at the participating centre during the reproductive years and according to the Rotterdam criteria [9].

Data collected in the registry are categorized into diagnostic data (age at diagnosis and diagnostic criteria used, including clinical hyperandrogenism [HA], biochemical HA, menstrual irregularities/oligoanovulation, and polycystic ovarian morphology [PCOm]); family history (cardiovascular or metabolic diseases, obesity, thyroid diseases, tumors, menstrual disorders, infertility, and hyperandrogenism); nutritional and physical activity habits; clinical characteristics (anthropometric measurements, blood pressure, hirsutism assessed by the modified Ferriman–Gallwey score (mFG) [10], acne, alopecia, comorbidities, and current medications); laboratory values (hormonal and metabolic parameters); gynecological data (menstrual history, pregnancy, fertility, and pelvic ultrasound); and psychological and sexual health, evaluated through validated questionnaires (Patient Health Questionnaire [PHQ-9], Generalized Anxiety Disorder 7-item scale [GAD-7], Female Sexual Function Index [FSFI], and Body Uneasiness Test [BUT] 4 A and B) [11–14]. Diagnostic data were referred to the time of diagnosis of PCOS, whereas all the other parameters (familiarity, nutritional and physical habits, clinical characteristics, laboratory values, gynecological data, and psychological and sexual behaviour) were recorded at the time of recruitment.

Data collection is based on standard-of-care examinations performed during diagnosis and routine clinical visits. The registry began enrolling patients prospectively in March 2020.

### Ethics approval and consent to participate

All centres participating in the PCOSOUTCOME.net registry obtained ethical approval from their respective medical ethics committees. All prospectively recruited patients gave informed consent.

### Study samples and parameters, imaging

For the current analysis, all Caucasian patients of reproductive age registered by November 2023 were included after plausibility checks and data harmonization procedures. Data plausibility checks were conducted centrally and included assessments of completeness as well as outlier detection. Centres were asked, where possible, to complete missing data or correct implausible entries.

We defined three study cohorts:

The first cohort was defined by PCOS phenotyping, from A to D, based on diagnostic data and the following criteria [15]:

A: clinical and or biochemical HA + menstrual irregularity/oligoanovulation + PCOm.

B: clinical and or biochemical HA + menstrual irregularity/oligoanovulation.

C: clinical and or biochemical HA + PCOm.

D: Menstrual irregularity or oligo-anovulation + PCOm.

The second cohort was classified based on BMI recorded at the time of recruitment into the following categories: normal-weight (18.5–24.9 kg/m²), overweight (25.0–29.9 kg/m²), and obesity (≥ 30 kg/m²), according to the WHO criteria for obesity.

The third cohort was defined by BMI recorded at the time of recruitment, with two groups: < 27 kg/m² and ≥ 27 kg/m². *This cut-off was chosen because recent publications have demonstrated that cardiometabolic risk increases at a BMI of 27 kg/m² or higher*,* at least in some populations* [16].

The diagnosis of PCOS was made without a current use of estrogen-progestin therapy and after excluding hyperprolactinemia, congenital adrenal hyperplasia, Cushing’s syndrome, androgen-secreting tumors, thyroid disorders, premature ovarian failure, or other specific causes of amenorrhea.

Clinical HA was defined at the presence of hirsutism, acne or androgenic alopecia. The mFG score was used to score hirsutism [10].

Biochemical HA was defined when total testosterone, androstenedione, or dehydroepiandrosterone sulphate (DHEA-S) levels were above the upper limit of normal range of the local laboratory, or with a calculated free androgen index (FAI) above 4.

Menstrual irregularity was defined according to reproductive age as the occurrence of menstrual cycles lasting more than 35 days or less than 21 days (≥ 3 years post-menarche), or cycles lasting more than 45 days or less than 21 days (1–3 years post-menarche). PCOm was defined as the presence of 10 or more follicles measuring 2–9 mm in diameter in the largest cross-sectional plane and/or an increased ovarian volume (> 10 mL) in at least one ovary [9].

*At the time of recruitment*,* patients had not received estrogen–progestin therapy*,* insulin-sensitizing agents*,* or any other medications known to affect steroid levels or glucose/insulin metabolism or action for at least 3 months prior to enrollment.*

For women with eumenorrhea or mild oligomenorrhea, blood samples for hormonal measurements were collected after an overnight fast during the first week following the start of spontaneous bleeding. Participants with moderate to severe oligomenorrhea or amenorrhea had blood samples taken at random after an overnight fast. Pelvic ultrasound was preferentially performed transvaginally, when possible during the follicular phase of the menstrual cycle.

Physical examinations at the time of recruitment included body measurements (height, weight, and waist circumference) and blood pressure. Patients were defined as hypertensive if any of the following applied: use of antihypertensive medication, systolic blood pressure ≥ 130 mmHg, or diastolic blood pressure ≥ 85 mmHg. Abdominal obesity was defined by a waist circumference ≥ 80 cm.

Dyslipidemia was defined as the presence of any of the following: use of hypolipidemic drugs, HDL cholesterol < 50 mg/dL, triglycerides ≥ 150 mg/dL, or LDL cholesterol ≥ 116 mg/dL.

Glucose tolerance status was estimated with both fasting glucose and glucose at 120 min from a 75 g oral glucose tolerance test (OGTT) (Curvosio, Sclavo, Cinisello Balsamo, Italy). The diagnosis of impaired fasting glucose (IFG), impaired glucose tolerance (IGT) or diabetes was performed according to American Diabetes Association criteria [17].

Metabolic syndrome was defined as the presence of at least three of the following criteria: abdominal obesity, diabetes or IFG or IGT, blood triglycerides ≥ 150 mg/dL, HDL cholesterol < 50 mg/dL, or hypertension [18].

Patients were defined as having insulin resistance if the Homeostasis Model Assessment of Insulin Resistance (HOMA-IR) index was ≥ 3 [19, 20].

### Statistical analyses

Descriptive statistical analyses were conducted, with continuous variables summarized as mean ± standard deviation and categorical variables expressed as percentages. Comparisons between two treatment groups were performed using the independent samples t-test or the Mann–Whitney U test, as appropriate. When more than two groups were evaluated, one-way analysis of variance (ANOVA) or the Kruskal–Wallis test was applied accordingly. Categorical variables were compared using the chi-square test or Fisher’s exact test, irrespective of the number of groups considered. A p-value < 0.05 was regarded as indicative of statistical significance. All statistical analyses were carried out using SAS (Version 9.4 - SAS Institute Inc.).

## Results

### Total study population

A total of 1314 Caucasian women of reproductive age with PCOS were enrolled in the registry (Table 1). Of these, *62*% had phenotype A, *20*% had phenotype B, *15*% had phenotype C, and *3*% had phenotype D. BMI data were available for 874 subjects; of these, *52*% was in the normal-weight, *20*% in the overweight, and *28*% in the obesity range. Among the 874 women with available BMI data, 351 (*40*%) had a BMI ≥ 27 kg/m². Systolic and diastolic blood pressure data were available for 449 women, and hypertension was identified in *31*% of them. Complete lipid profiles were available for 459 women, and dyslipidemia was observed in *55*%. Insulin resistance, assessed in 637 patients, had a prevalence of *29*%. Glucose tolerance was evaluated in 390 PCOS patients with available fasting glucose and 120-minute post-OGTT glucose values. In this cohort, the prevalence of glucose intolerance was *15*%, with *14*% having IFG and/or IGT and *1*% having diabetes. Metabolic syndrome, estimated in the subgroup with complete data for all parameters included in its definition (*n* = 176 PCOS), affected *46*% of the population.

**Table 1:** General characteristics of the Caucasian population with Polycystic Ovary Syndrome (*n* = 1,314)

Variables	Mean ± SD or number (%)
Age (years)	23 ± 6
PCOS phenotypes:	
A	813 (62%)
B	269 (20%)
C	196 (15%)
D	36 (3%)
BMI (kg/m^2^); *n* = 874	26 ± 7
BMI categories:	
Normal-weight	451 (52%)
Overweight	176 (20%)
Obesity	247 (28%)
Waist circumference (cm); *n* = 614	86 ± 16
Abdominal phenotype, Yes	349 (57%)
Blood pressure (mm/Hg)	
Systolic; *n* = 490	119 ± 13
Diastolic; *n* = 449	77 ± 10
Hypertension, Yes; *n* = 449*	139 (31%)
Lipid profile (mg/dL)	
Total cholesterol; *n* = 481	173 ± 34
HDL cholesterol; *n* = 470	55 ± 15
LDL cholesterol; *n* = 460	100 ± 28
Triglycerides; *n* = 472	84 ± 53
Dyslipidemia, Yes; *n* = 459*	252 (55%)
Glucose, fasting (mg/dL); *n* = 780	85 ± 12
Glucose, 120 min OGTT (mg/dL); *n* = 427	103 ± 43
Insulin, fasting (µU/mL); *n* = 660	12 ± 8
HOMA-IR; *n* = 637	2.6 ± 1.9
Insulin resistance, Yes; *n* = 637	183 (29%)
Glucose intolerance, Yes; *n* = 390*	59 (15%)
IFG and/or IGT	54 (14%)
Diabetes	5 (1%)
Metabolic syndrome, Yes; *n* = 176*	81 (46%)
Questionnaires:	
PHQ-9, Positive; *n* = 98	32 (33%)
Slight	21 (21%)
Moderate	9 (9%)
Severe	2 (2%)
GAD7, Positive; *n* = 97	69 (71%)
Slight	46 (47%)
Moderate	17 (17%)
Severe	6 (6%)
BUT4, *n* = 112	
A, GSI, Positive	59 (53%)
B, PST	14 ± 9
B, PSDI	2 ± 1
FSFI, Positive; *n* = 131	90 (69%)

PCOS: polycystic ovary syndrome; BMI: body mass index; OGTT: oral glucose tolerance test; HOMA-IR: Homeostatic Model Assessment for Insulin Resistance; IFG: impaired fasting glucose; IGT: impaired glucose tolerance; PHQ-9: Patient Health Questionnaire; GAD7: Generalized Anxiety Disorder 7-item scale; BUT4; Body Uneasiness Test; GSI; Global Severity Index; PST: Positive Symptom Total; PSDI: Positive Symptom Distress Index; FSFI: Female Sexual Function Index

*Prevalence of hypertension, dyslipidemia, glucose intolerance, and metabolic syndrome was calculated in the population with complete data for all parameters included in the definition of the metabolic disorder

Questionnaires assessing anxiety, depression, body image distress, and sexual symptoms were administered to a subcohort of patients (Table 1). Anxiety, evaluated using the GAD-7 questionnaire in 97 patients, was identified in *71*%. Depression, assessed with the PHQ-9 questionnaire in 98 patients, was detected in *33*%. Body image distress, measured using the BUT4A questionnaire in 112 patients, was present in *53*%. A pathological total score on the FSFI, indicative of sexual symptoms, was observed in *69*% of patients.

### Four subpopulations by Rotterdam criteria

The four PCOS phenotypes (A-D) had similar BMI, waist circumference, systolic and diastolic blood pressure, as well as a comparable prevalence of overweight and obesity, abdominal obesity, hypertension, dyslipidemia, glucose intolerance, and metabolic syndrome (Table 2). No significant differences were found in fasting glucose, fasting insulin, or HOMA-IR levels among the phenotypes; however, the prevalence of insulin resistance was higher in phenotypes A and D (Table 2). Among the four psychological questionnaires analyzed, only the GAD-7 showed a significant difference across the PCOS phenotypes. Phenotype A had the highest anxiety scores, whereas phenotype D had the lowest (Table 2).

**Table 2:** Clinical and biochemical parameters in the four PCOS phenotypes

Variables	A phenotype (*n* = 813)	B phenotype (*n* = 269)	C phenotype (*n* = 196)	D phenotype (*n* = 36)	*P* value
Age (years)	23 ± 6	23 ± 7	23 ± 6	25 ± 6	**0.044**
BMI (kg/m^2^)	26 ± 7 (*n* = 555)	27 ± 7 (*n* = 176)	26 ± 6 (*n* = 128)	26 ± 6 (*n* = 15)	0.735
BMI categories:					0.330
Normal-weight	290 (52%)	82 (47%)	70 (55%)	9 (60%)	
Overweight	101 (18%)	45 (26%)	28 (22%)	2 (13%)	
Obesity	164 (29%)	49 (28%)	30 (23%)	4 (27%)	
BMI ≥ 27 kg/m²	222 (40%)	77 (44%)	46 (36%)	6 (40%)	0.594
Waist circumference (cm)	85 ± 17 (*n* = 417)	87 ± 16 (*n* = 113)	84 ± 13 (*n* = 77)	82 ± 20 (*n* = 7)	0.529
Abdominal phenotype	232 (56%)	70 (62%)	44 (57%)	3 (43%)	0.582
Blood pressure (mm/Hg):					
Systolic	119 ± 13(*n* = 334)	120 ± 15(*n* = 81)	119 ± 13(*n* = 69)	113 ± 10(*n* = 6)	0.729
Diastolic	76 ± 9(*n* = 307)	78 ± 11(*n* = 70)	75 ± 10(*n* = 67)	80 ± 10(*n* = 5)	0.266
Hypertension*	89 (29%)	27 (39%)	21 (31%)	2 (40%)	0.408
Lipid profile (mg/dL):					
Total cholesterol	173 ± 34(*n* = 322)	176 ± 34(*n* = 92)	160 ± 31(*n* = 60)	177 ± 24(*n* = 7)	**0.013**
HDL cholesterol	56 ± 16(*n* = 317)	54 ± 12(*n* = 89)	52 ± 10(*n* = 57)	65 ± 19(*n* = 7)	0.099
LDL cholesterol	100 ± 28(*n* = 312)	106 ± 31(*n* = 87)	93 ± 27(*n* = 54)	98 ± 26(*n* = 7)	0.115
Triglycerides	87 ± 57(*n* = 317)	81 ± 37(*n* = 90)	78 ± 45(*n* = 58)	73 ± 45(*n* = 7)	0.669
Dyslipidemia*	162 (52%)	53 (61%)	33 (61%)	4 (57%)	0.386
Glucose, fasting (mg/dL)	85 ± 13 (*n* = 488)	85 ± 11 (*n* = 158)	86 ± 9 (*n* = 121)	84 ± 10 (*n* = 13)	0.426
Glucose, 120 min OGTT (mg/dL)	104 ± 33 (*n* = 285)	109 ± 76 (*n* = 74)	95 ± 24 (*n* = 62)	103 ± 37 (*n* = 6)	0.290
Insulin, fasting (µU/mL)	12 ± 9 (*n* = 422)	11 ± 8 (*n* = 129)	10 ± 7 (*n* = 99)	13 ± 9 (*n* = 10)	0.154
HOMA-IR	2.7 ± 2.0 (*n* = 406)	2.5 ± 1.9 (*n* = 127)	2.2 ± 1.6 (*n* = 95)	2.6 ± 2.0 (*n* = 9)	0.205
Insulin resistance	130 (32%)	34 (27%)	16 (17%)	3 (33%)	**0.029**
Glucose intolerance*:					0.330
IFG and/or IGT	37 (14%)	12 (19%)	4 (7%)	1 (17%)	
Diabetes	3 (1%)	2 (3%)	0 (0%)	0 (0%)	
Metabolic syndrome*	61 (45%)	11 (65%)	8 (38%)	1 (50%)	0.390
Questionnaires:					
PHQ9, Positive	15 (35%)	12 (41%)	5 (21%)	0 (0%)	0.362
GAD7, Positive	35 (81%)	20 (69%)	14 (61%)	0 (0%)	**0.043**
BUT,					
A, GSI, Positive	33 (55%)	15 (58%)	11 (44%)	0 (0%)	0.547
B, PST	14 ± 8	14 ± 9	15 ± 11	14	0.995
B, PSDI	2 ± 1	2 ± 1	2 ± 1	1	0.389
FSFI, Positive	45 (66%)	24 (69%)	19 (73%)	2 (100%)	0.901

PCOS: polycystic ovary syndrome; BMI: body mass index; OGTT: oral glucose tolerance test; HOMA-IR: Homeostatic Model Assessment for Insulin Resistance; IFG: impaired fasting glucose; IGT: impaired glucose tolerance; PHQ-9: Patient Health Questionnaire; GAD7: Generalized Anxiety Disorder 7-item scale; BUT4; Body Uneasiness Test; GSI; Global Severity Index; PST: Positive Symptom Total; PSDI: Positive Symptom Distress Index; FSFI: Female Sexual Function Index

*Prevalence of hypertension, dyslipidemia, glucose intolerance, and metabolic syndrome was calculated in the population with complete data for all parameters included in the definition of the metabolic disorder

Data are reported as mean ± SD or number (%)

### Three subpopulations by BMI

When the study sample was analyzed according to the three BMI categories (normal-weight, overweight, and obesity), significant differences appeared in all the metabolic measurements, except for fasting glucose, which was similar among the three groups (Table 3). In particular, waist circumference, systolic and diastolic blood pressure, lipid profile, glucose levels at 120 min of the OGTT, fasting insulin, and HOMA-IR were *more unfavorable* in the obesity group and *more favorable* in the normal-weight group. Similarly, the highest prevalence of abdominal obesity, hypertension, dyslipidemia, insulin resistance, glucose intolerance, and metabolic syndrome was reported in the obesity group, followed by the overweight and normal-weight groups (Table 3). The four questionnaires administered (PHQ-9, GAD-7, BUT4A and B, and FSFI) did not differ among the three BMI categories (Table 3). Hirsutism had the highest score in the obesity and the lowest score in the normal-weight group, whereas the prevalence of menstrual abnormalities and PCOm on pelvic ultrasound did not differ among the three BMI categories (Table 3). LH, FSH, and LH-to-FSH ratio were similar among the three BMI categories, as well as total testosterone, androstenedione, and DHEA-S. SHBG had the lowest values in the obesity group and the highest values in the normal-weight group; consequently, the FAI was highest in the obesity group and lowest in the normal-weight group (Table 3).

**Table 3:** Clinical and biochemical parameters in the three BMI classes

Variables	Normal-weight (*n* = 451)	Overweight (*n* = 176)	Obesity (*n* = 247)	*P* value
Age (years)	22 ± 6	23 ± 6	24 ± 7	**0.002**
BMI ≥ 27 kg/m²	0 (0%)	104 (59%)	247 (100%)	**< 0.0001**
BMI < 27 kg/m²	451 (100%)	72 (41%)	0 (0%)	
Waist circumference (cm)	73 ± 8 (*n* = 287)	88 ± 9 (*n* = 126)	103 ± 11 (*n* = 190)	**< 0.0001**
Abdominal phenotype	51 (18%)	103 (82%)	190 (100%)	**< 0.0001**
Blood pressure (mm/Hg):				
Systolic	115 ± 12(*n* = 214)	116 ± 12(*n* = 99)	127 ± 13(*n* = 163)	**< 0.0001**
Diastolic	73 ± 8(*n* = 188)	74 ± 9(*n* = 94)	81 ± 9(*n* = 153)	**< 0.0001**
Hypertension*	26 (14%) (*n* = 188)	20 (21%) (*n* = 94)	88 (57%) (*n* = 153)	**< 0.0001**
Lipid profile (mg/dL): Total cholesterol HDL cholesterol LDL cholesterol Triglycerides	168 ± 31 (*n* = 208) 61 ± 15 (*n* = 201) 94 ± 25 (*n* = 197) 65 ± 32 (*n* = 205)	171 ± 36 (*n* = 97) 52 ± 12 (*n* = 96) 102 ± 31 (*n* = 91) 82 ± 46 (*n* = 92)	179 ± 35 (*n* = 151) 49 ± 12 (*n* = 149) 108 ± 29 (*n* = 148) 111 ± 64 (*n* = 151)	**0.005** **< 0.0001** **< 0.0001** **< 0.0001**
Dyslipidemia*	70 (36%) (*n* = 197)	64 (68%) (*n* = 90)	111 (75%) (*n* = 148)	**< 0.0001**
Glucose, fasting (mg/dL)	85 ± 13 (*n* = 352)	85 ± 10 (*n* = 152)	85 ± 11 (*n* = 218)	0.93
Glucose, 120 min OGTT (mg/dL)	97 ± 53 (*n* = 174)	101 ± 31 (*n* = 90)	115 ± 35 (*n* = 136)	**0.0007**
Insulin, fasting (µU/mL)	9 ± 6 (*n* = 294)	12 ± 8 (*n* = 128)	17 ± 9 (*n* = 193)	**< 0.0001**
HOMA-IR	1.8 ± 1.4 (*n* = 281)	2.4 ± 1.6 (*n* = 123)	3.8 ± 2.2 (*n* = 190)	**< 0.0001**
Insulin resistance	39 (14%)	31 (25%)	105 (55%)	**< 0.0001**
Glucose intolerance*: IFG and/or IGT Diabetes	12 (7%) 2 (1%) *n* = 159	15 (17%) 1 (1%) *n* = 86	23 (19%) 2 (2%) *n* = 123	**0.010**
Metabolic syndrome*	6 (11%) *n* = 53	12 (33%) *n* = 36	61 (73%) *n* = 84	**< 0.0001**
Questionnaires:				
PHQ9, Positive	11 (31%)*n* = 36	8 (36%)*n* = 22	10 (32%)*n* = 31	0.90
GAD7, Positive	25 (71%)*n* = 35	16 (73%)*n* = 22	21 (68%)*n* = 31	0.91
BUT4				
A, GSI, Positive	18 (40%)*n* = 45	16 (62%)*n* = 26	21 (58%)*n* = 36	0.13
B, PST	14 ± 9	14 ± 8	15 ± 8	0.74
B, PSDI	2 ± 1	2 ± 1	2 ± 1	0.75
FSFI, Positive	38 (68%)*n* = 56	23 (72%)*n* = 32	25 (64%)*n* = 39	0.78
mFG score	9 ± 6	10 ± 7	11 ± 6	**0.001**
Menstrual irregularities	382 (85%)	150 (85%)	217 (88%)	0.44
PCOm	370 (82%)	132 (75%)	198 (80%)	0.12
LH (mU/mL)	9 ± 6	9 ± 6	8 ± 5	0.37
FSH (mU(mL)	6 ± 2	6 ± 2	5 ± 2	0.10
LH-to-FSH ratio	2 ± 1	2 ± 1	2 ± 1	0.85
Total testosterone (ng/mL)	0.6 ± 0.3	0.6 ± 0.3	0.6 ± 0.3	0.32
SHBG (nmol/L)	56 ± 27	40 ± 18	31 ± 24	**< 0.0001**
FAI	1 ± 1	2 ± 2	3 ± 3	**< 0.0001**
Androstenedione (ng/dL)	338 ± 178	355 ± 189	357 ± 177	0.40
DHEA-S (µg/mL)	478 ± 242	525 ± 238	522 ± 261	0.10

PCOS: polycystic ovary syndrome; BMI: body mass index; OGTT: oral glucose tolerance test; HOMA-IR: Homeostatic Model Assessment for Insulin Resistance; IFG: impaired fasting glucose; IGT: impaired glucose tolerance; PHQ-9: Patient Health Questionnaire; GAD7: Generalized Anxiety Disorder 7-item scale; BUT4; Body Uneasiness Test; GSI; Global Severity Index; PST: Positive Symptom Total; PSDI: Positive Symptom Distress Index; FSFI: Female Sexual Function Index; mFG: modified Ferriman-Gallwey; PCOm: polycystic ovarian morphology; LH: luteinizing hormone; FSH: follicle-stimulating hormone; SHBG: sex hormone-binding globulin; FAI: free androgen index; DHEA-S: dehydroepiandrosterone sulphate

*Prevalence of hypertension, dyslipidemia, glucose intolerance, and metabolic syndrome was calculated in the population with complete data for all parameters included in the definition of the metabolic disorder

Data are reported as mean ± SD or number (%)

### Two subpopulations by BMI

Similar results to those identified in the three BMI classes were obtained by subdividing the PCOS cohort into two groups according to BMI < 27 kg/m² or ≥ 27 kg/m² (Table 4). In particular, the group with BMI ≥ 27 kg/m² had higher waist circumference, systolic and diastolic blood pressure, total cholesterol, LDL cholesterol, triglycerides, glucose levels at 120 min of the OGTT, fasting insulin, HOMA-IR, FAI, and mFG score compared to the group with BMI < 27 kg/m². HDL cholesterol and SHBG were lower in the group with BMI ≥ 27 kg/m². The prevalence of abdominal obesity, hypertension, dyslipidemia, insulin resistance, and metabolic syndrome was highest in the group with BMI ≥ 27 kg/m², whereas the prevalence of glucose intolerance states showed borderline significance.

**Table 4:** Clinical and biochemical parameters in the PCOS group with BMI < 27 kg/m² vs. the PCOS group with BMI ≥ 27 kg/m²

Variables	BMI< 27 kg/m^2^ (*n* = 523)	BMI ≥ 27 kg/m² (*n* = 351)	*P* value
Age (years)	22 ± 6	24 ± 7	**0.0005**
Waist circumference (cm)	75 ± 9 (*n* = 339)	100 ± 12 (*n* = 264)	**< 0.0001**
Abdominal phenotype	87 (26%)	257 (97%)	**< 0.0001**
Blood pressure (mm/Hg): Systolic Diastolic	115 ± 12 (*n* = 249) 74 ± 9 (*n* = 220)	124 ± 13 (*n* = 227) 79 ± 10 (*n* = 215)	**< 0.0001** **< 0.0001**
Hypertension*	33 (15%) (*n* = 220)	101 (47%) (*n* = 215)	**< 0.0001**
Lipid profile (mg/dL): Total cholesterol HDL cholesterol LDL cholesterol Triglycerides	168 ± 32 (*n* = 246) 59 ± 15 (*n* = 239) 95 ± 26 (*n* = 231) 67 ± 34 (*n* = 240)	178 ± 35 (*n* = 210) 49 ± 12 (*n* = 207) 107 ± 30 (*n* = 205) 104 ± 61 (*n* = 208)	**0.002** **< 0.0001** **< 0.0001** **< 0.0001**
Dyslipidemia*	92 (39%) (*n* = 230)	152 (74%) (*n* = 205)	**< 0.0001**
Glucose, fasting (mg/dL)	85 ± 13 (*n* = 410)	85 ± 10 (*n* = 312)	0.71
Glucose, 120 min OGTT (mg/dL)	97 ± 50 (*n* = 210)	112 ± 34 (*n* = 190)	**0.0005**
Insulin, fasting (µU/mL)	9 ± 6 (*n* = 346)	16 ± 9 (*n* = 269)	**< 0.0001**
HOMA-IR	1.9 ± 1.3 (*n* = 330)	3.5 ± 2.1 (*n* = 264)	**< 0.0001**
Insulin resistance	46 (14%)	129 (49%)	**< 0.0001**
Glucose intolerance*: IFG and/or IGT Diabetes	19 (10%) 3 (2%) *n* = 191	31 (17%) 2 (1%) *n* = 177	**0.06**
Metabolic syndrome*	11 (17%) *n* = 65	68 (63%) *n* = 108	**< 0.0001**
Questionnaires: PHQ9, Positive GAD7, Positive BUT4 A, GSI, Positive B, PST B, PSDI FSFI, Positive	13 (32%) *n* = 41 29 (72%) *n* = 40 25 (47%) *n* = 53 14 ± 9 2 ± 1 44 (67%) *n* = 65	16 (33%) *n* = 48 33 (69%) *n* = 48 30 (56%) *n* = 54 15 ± 8 2 ± 1 42 (67%) *n* = 62	0.87 0.70 0.39 0.64 0.57 1.00
mFG score	10 ± 6	11 ± 6	**0.030**
Menstrual irregularities	443 (85%)	306 (87%)	0.66
PCOm	426 (81%)	274 (78%)	0.28
LH (mU/mL)	9 ± 6	8 ± 5	0.44
FSH (mU(mL)	6 ± 2	5 ± 2	0.06
LH-to-FSH ratio	2 ± 1	2 ± 1	0.67
Total testosterone (ng/mL)	1 ± 0	1 ± 0	0.45
SHBG (nmol/L)	54 ± 26	34 ± 23	**< 0.0001**
FAI	1 ± 1	3 ± 3	**< 0.0001**
Androstenedione (ng/dL)	341 ± 178	357 ± 183	0.25
DHEA-S (µg/mL)	482 ± 240	526 ± 258	**0.040**

PCOS: polycystic ovary syndrome; BMI: body mass index; OGTT: oral glucose tolerance test; HOMA-IR: Homeostatic Model Assessment for Insulin Resistance; IFG: impaired fasting glucose; IGT: impaired glucose tolerance; PHQ-9: Patient Health Questionnaire; GAD7: Generalized Anxiety Disorder 7-item scale; BUT4; Body Uneasiness Test; GSI; Global Severity Index; PST: Positive Symptom Total; PSDI: Positive Symptom Distress Index; FSFI: Female Sexual Function Index; mFG: modified Ferriman-Gallwey; PCOm: polycystic ovarian morphology; LH: luteinizing hormone; FSH: follicle-stimulating hormone; SHBG: sex hormone-binding globulin; FAI: free androgen index; DHEA-S: dehydroepiandrosterone sulphate

*Prevalence of hypertension, dyslipidemia, glucose intolerance, and metabolic syndrome was calculated in the population with complete data for all parameters included in the definition of the metabolic disorder

Data are reported as mean ± SD or number (%)

## Discussion

This is the first cross-sectional observational study performed on an Italian PCOS registry comparing the ability of the Rotterdam phenotypic classification and BMI to characterize phenotypic heterogeneity in women with PCOS, with particular attention to cardiometabolic parameters.

Interestingly, we found no significant differences in metabolic parameters or in the prevalence of hypertension, dyslipidemia, glucose intolerance, and metabolic syndrome among the four PCOS phenotypes (A–D).

In contrast, we observed significant differences among the three BMI classes. In particular, the group with the *least favorable* metabolic profile and the highest prevalence of hypertension, dyslipidemia, glucose intolerance, and metabolic syndrome was the obesity group, followed by the overweight and normal-weight groups.

Similar results to those observed across the three BMI classes were obtained when the PCOS cohort was subdivided into two groups according to BMI < 27 kg/m² or ≥ 27 kg/m². In particular, the prevalence of hypertension, dyslipidemia, and metabolic syndrome was highest in the group with BMI ≥ 27 kg/m², whereas the prevalence of glucose intolerance lost statistical significance, reaching only borderline significance. In addition, the highest prevalence of abdominal obesity and insulin resistance was observed in the obesity group and in the group with BMI ≥ 27 kg/m².

Our findings that cardiometabolic parameters were comparable across the four PCOS phenotypes appears to contrast with some previous studies reporting a more adverse metabolic profile in hyperandrogenic phenotypes, although the available evidence remains inconsistent [21–23], particularly after accounting for differences in BMI or adiposity. Indeed, in some studies, adjustment for BMI or fat mass abolished these differences [8, 24], whereas in others they persisted [22, 25]. These discrepancies may reflect residual confounding related to adiposity, variability in the assessment of hyperandrogenism and metabolic parameters, as well as differences in study design and population characteristics. While clinical trials ensure high internal validity, real-world cohort studies provide a complementary perspective by capturing patient heterogeneity and disease variability in routine clinical practice, thereby enhancing the generalizability of findings. Additionally, the relatively small number of patients with phenotype D recruited in this observational study, despite the large overall cohort, may have limited the statistical power to detect differences among phenotypes.

Overall, this study adds to the growing body of evidence suggesting that, although androgen excess may contribute to metabolic dysfunction, its impact appears less pronounced than that of obesity [24, 26]. BMI ≥ 30 kg/m² and BMI ≥ 27 kg/m² appear to have similar predictive value for the risk of metabolic alterations, except for glucose intolerance, for which BMI ≥ 30 kg/m² seems to be more predictive. These results underscore the critical importance of identifying moderate overweight in the metabolic evaluation of patients with PCOS. Indeed, a BMI threshold of 27 appears to represent a “tipping point” for metabolic risk, with important implications for clinical management, particularly with regard to preventive strategies and lifestyle interventions.

Interestingly, and in agreement with a previous study [24], the obese PCOS subgroup exhibited the highest levels of free testosterone, estimated by the FAI, and the lowest levels of SHBG, while circulating concentrations of total testosterone, androstenedione, and DHEA-S were similar to those observed in both the overweight and normal-weight groups. Likewise, LH, FSH, and the LH-to-FSH ratio did not differ across the three BMI categories. Hirsutism scores were highest in the obesity group and lowest in the normal-weight group, whereas the prevalence of menstrual abnormalities and PCOm did not differ among BMI categories.

These findings support the notion that obesity identifies a distinct hyperandrogenic phenotype in PCOS, characterized by a selective increase in circulating free testosterone and more severe hirsutism, likely driven by reduced SHBG levels. This reduction may reflect the combined effects of insulin resistance and excess adiposity.

Unexpectedly, differences in psychological health questionnaires were identified only among the four Rotterdam phenotypes, but not among different BMI subgroups. In particular, Rotterdam phenotype A showed the highest anxiety scores. PCOS is notoriously associated with body image distress [27–29], with hyperandrogenism, body weight and reproductive disturbances among the main drivers [27, 30], resulting in higher risk of anxiety, depression and sexual dysfunction [27–29, 31]. Our findings partly corroborate these results, with anxiety being more prevalent in patients displaying both hyperandrogenism and menstrual dysfunction. On the other hand, surprisingly, BMI did not seem to influence psychological health in our patients. This result, however, may have been influenced by a lower number of completed questionnaires.

*We recognize that a limitation of this study is that adiposity was not assessed using direct measurements of body fat*,* such as dual-energy X-ray absorptiometry (DEXA) or bioimpedance analysis* [32]. *In addition*,* we did not measure hip circumference; therefore*,* we were unable to provide information on an important clinical parameter*,* namely the waist-to-hip ratio.*

In conclusion, this study confirms that obesity is the primary driver of metabolic disorders in PCOS. This perspective, although requiring cautious interpretation, may have important implications for metabolic assessment and risk stratification in women with PCOS, particularly in light of the broad spectrum of clinical features in this population. Overall, these findings support the integration of BMI into the classification of PCOS to better capture patient heterogeneity.
